# Why do pregnant women in Iringa region in Tanzania start antenatal care late? A qualitative analysis

**DOI:** 10.1186/s12884-020-2823-4

**Published:** 2020-02-24

**Authors:** Stephen Oswald Maluka, Chakupewa Joseph, Sian Fitzgerald, Robert Salim, Peter Kamuzora

**Affiliations:** 1 Institute of Development Studies, University of Dar es Salaam, P.O.BOX 35169, Dar es Salaam, Tanzania; 2 Mkwawa University College of Education (MUCE), P.O.BOX 2515, Iringa, Tanzania; 3 Healthbridge Foundation of Canada, Ottawa, Canada; 4 Iringa Regional Commissioner’s Office, Health Department, Iringa, Tanzania

**Keywords:** Antenatal care, Late attendance, Tanzania

## Abstract

**Background:**

When started early in pregnancy and continued up till childbirth, antenatal care (ANC) can be effective in reducing adverse pregnancy outcomes. While the proportion of women who attend ANC at least once in low income countries is high, most pregnant women attend their first ANC late. In Tanzania, while over 51% of pregnant women complete ≥4 visits, only 24% start within the first trimester. This study aimed to understand the factors that lead to delay in seeking ANC services among pregnant women in Tanzania.

**Methods:**

This qualitative descriptive case study was conducted in two rural districts in Iringa Region in Tanzania. A total of 40 focus group discussions (FGDs) were conducted involving both male and female participants in 20 villages. In addition, 36 semi-structured interviews were carried out with health care workers, members of health facility committees and community health workers. Initial findings were further validated during 10 stakeholders’ meetings held at ward level in which 450 people participated. Data were analysed using thematic approach.

**Results:**

Key individual and social factors for late ANC attendance included lack of knowledge of the importance of early visiting ANC, previous birth with good outcome, traditional gender roles, fear of shame and stigma, and cultural beliefs about pregnancy. Main factors which inhibit early ANC attendance in Kilolo and Mufindi districts include spouse accompany policy, rude language of health personnel and shortage of health care providers.

**Conclusions:**

Traditional gender roles and cultural beliefs about pregnancy as well as health system factors continue to influence the timing of ANC attendance. Improving early ANC attendance, therefore, requires integrated interventions that address both community and health systems barriers. Health education on the timing and importance of early antenatal care should also be strengthened in the communities. Additionally, while spouse accompany policy is important, the implementation of this policy should not infringe women’s rights to access ANC services.

## Introduction

Every day, approximately 830 women die from preventable causes related to pregnancy and childbirth while 99% of all maternal deaths occur in low- and middle-income countries (LMICs) [1]. When started early in pregnancy and continued up till childbirth, antenatal care (ANC) can effectively reduce adverse pregnancy outcomes. It has been established that about 25% of maternal deaths occur during the prenatal period, caused mainly by (pre-)eclampsia and antepartum haemorrhage that are manageable, if pregnant women attend ANC on time [2]. The World Health Organization (WHO) recommends that a pregnant woman without complications attend ANC at least eight times; and that the first attendance takes place within the first trimester [3].

ANC services provide a platform for critical healthcare functions, including health care promotion and prevention, screening and diagnosis of diseases [4]. This provides opportunities to detect and treat complications of pregnancy early, and ensures preventive interventions such as tetanus toxiod immunisation, intermittent preventive treatment of malaria, deworming, iron and folic acid prescriptions and insecticide-treated bed nets [5]. Studies show further that women who attend ANC promptly have a high chance to access skilled birth attendants during birth [6, 7]. Above all, when expectant mothers and their spouses attend ANC together, they get exposed to health education on danger signs and birth preparedness. Couples are also educated on several topics, including nutrition, family planning, effective breastfeeding, lifestyle, exercise, personal and environmental hygiene and safety [8, 9].

Although the percentage pregnant women who attend ANC at least once in LMICs is high, most of them attend their first ANC late [10–12]. For instance, in Ethiopia in 2014, only 18% of pregnant women attended ANC early with 32% attending four times as recommended [10]. In Uganda, only 21% attended ANC early with 48% attending four times [11]. In Tanzania, while over 90% of pregnant women attend ANC at least once, 51% attended ≥4 times and 24% attended the first ANC-visit before the fourth month of pregnancy [12].

Studies have reported a number of possible reasons for delayed ANC attendance; the most cited being socio-demographic attributes [13–16]. For instance, it has been reported that educated women married to educated men are more likely to attend ANC earlier than the less educated counterparts [14, 15]. Similarly, younger women are likely to attend ANC later than older ones [16]. Moreover, the quality of ANC like technical competency of service providers may determine ANC service uptake [17, 18]. For instance, where women perceive the care as sub standard coupled with negative attitudes toward health care providers, women would shun health care at such facilities [19]. The costs associated with ANC service are also reported to contribute to late ANC attendance [20]. The costs may be direct or indirect, and include payment for laboratory tests, transport, food, company and maternity attire [20]. Studies have also reported unwanted pregnancies as among the factors accounting for delayed ANC attendance [21–25].

Qualitative studies have highlighted the importance of understanding context-specific factors for ANC attendance [16–19, 26–28]. In order to inform the design and implementation of interventions to improve early ANC attendance, this study adopted a qualitative approach to explore factors leading to delayed ANC attendance in Iringa Region in Tanzania.

## Methods

### Study design and settings

A descriptive case study design was used that is suitable to investigate a phenomenon in its real life setting [29]. This study is part of the baseline assessment of the Innovating for Maternal and Child Health in Africa (IMCHA) programme which is being implemented in Iringa Region, Tanzania by the Institute of Development Studies, University of Dar es Salaam in collaboration with Iringa Region Health Department and Health Bridge Foundation of Canada. Iringa Region was selected because of earlier collaboration between some of the IMCHA researchers and the regional and district decision makers on strengthening decentralised district health management. Like in other rural districts in Tanzania, ANC indicators in Iringa Region are low. The proportions of pregnant women who attend ANC within the first trimester were 27% in Kilolo and 17% in Mufindi district [30].

### Data collection

A total of 40 Focus Group Discussions (FGDs) were conducted in 20 villages of Kilolo and Mufindi districts. The villages were selected purposively because they were part of the IMCHA project. Each FGD consisted of 10–12 participants; and females and males had separate sessions in each village. FGD participants were selected purposively in collaboration with the community health workers. The criteria for selecting female participants included: age (15–49 years), experience in utilizing ANC services, and pregnancy or birth in the last 12 months preceding the study. The involved male participants were required to be either married or otherwise live with a female partner and have experience with childbirth. All FGDs were conducted in a private room in the village offices and digitally recorded with permission from the participants. FGD guides were developed and used to guide the discussion (Additional file 1). FGDs were conducted in 2016 and each FGD session lasted for between 45 min and one hour. FGDs were facilitated by trained female and male researchers. In addition, we conducted semi-structured interviews with health care workers. A copy of the interview has been included as supplementary file (Additional file 2). Interviewees were purposively selected based on the active roles in the provision and management of maternal and child health services. In total, 80 interviews were carried out; an average of four interviews for each village. Saturation point was reached when no any new information was coming out of the interviews. Interviews were conducted by SM, CJ and PK in Kiswahili language in 2016. All interviews were recorded after getting permission from the respondents. Furthermore, initial findings of the study were validated during stakeholders’ meetings which were held in all 10 Wards. The stakeholders’ meetings involved male and female champions, community and religious leaders, health care providers and members of the user committees. The meetings were conducted between January and March 2018, and the number of participants in each meeting ranged from 40 to 50. The stakeholders’ meetings were coordinated by the researchers involved in the implementation of the IMCHA project.

### Data analysis

All FGDs and interviews were transcribed by trained transcribers. The transcripts were reviewed by the core research team members and notes were made for each transcript. A thematic approach [31] was used to analyse FGDs, interviews, and summaries of the stakeholders’ meetings. An initial coding framework was developed by the Principal Investigator (SM) based on the objectives of the study. The coding manual was further discussed and refined by the research team members. NVivo10 qualitative data analysis software was used for coding and managing data [32]. Two members of the research team (SM, CJ) independently coded the first five interviews to ensure consistency. Thereafter, SM and CJ continued to code the transcripts and summaries of the meetings. Responses were compared across different types of respondents and across the two districts. Finally, data were summarized and synthesized and key terms and phrases of respondents were used to support findings.

## Results

The key themes that emerged are: lack of knowledge and past experience with safe delivery, traditional gender roles, shame and stigma, superstitions, partner accompany policy, rude language and shortage of health care workers.

### Lack of knowledge and previous birth with good outcome

Some women and men did not know the importance of attending ANC early during pregnancy. Since inexperienced women and men were not familiar with the signs of pregnancy, they could not discover or disclose pregnancy early enough. Similarly, students and unmarried young women fear to disclose pregnancy for fear of expulsion from school and from the family as well as abandonment by male partners. Furthermore, it was reported in most FGDs and confirmed during stakeholders’ meetings that women who had previously given birth with good outcome do not see the importance of attending ANC early.

During the stakeholders’ meeting, many participants had the same view regarding the challenges about experience with safe delivery:

*It has been a tendency in this village for pregnant women to attend ANC very late. Sometimes I speak with women at my church. It is astonishing to hear, “I gave birth to my last born very easily; I only attended ANC once in the fifth month of pregnancy, and my daughter is healthy. As a man of God, when you hear that you start realizing how big the problem is* (Religious Leader during stakeholders’ meeting).

### Traditional gender roles

Female participants demonstrated awareness that couples should attend ANC together. It was further learnt that in most cases, pregnant women initiate the need to attend ANC. However, men have high decision-making power to approve or disapprove women’s proposal to attend ANC. As a result, women’s decision to attend ANC early was perceived to be limited. Women in the FGDs reported that men control almost everything, including farms, livestock and businesses. In some cases, women could not attend ANC early due to lack of fare and other necessary needs related to maternity.

Men were perceived as breadwinners and their main role was to support their partners financially. Relevant to ANC attendance, men were expected to be responsible for birth preparedness and complication readiness. According to our participants, men who attend ANC with their wives were perceived to be dominated by women, and this was shameful. During stakeholders’ meetings, female participants reported that even when their spouses attended ANC with them, they were ridiculed for engaging in traditionally defined women’s work.

### Fear of stigma

Families in Kilolo and Mufindi districts face the problem of short birth spacing, referred to as “Kufudikira” in Hehe and Bena languages. This was not acceptable in the family, community and by health care providers. As a result, once women realize that they are pregnant within a short time after the last birth, they delay ANC attendance. FGD’s participants indicated that women were afraid to be scorned if health care providers learnt they left babies under six months at home. Nurses and doctors tend then to shout with loud voices such that they are heard even outside the consultation room. This was also confirmed in an interview with a member of a health facility committee who said that;

*Men always do not feel comfortable to attend ANC with their partners who have little babies because they will be stigmatised by their fellow men and health care providers. This makes women delay ANC while waiting for the children to grow* (Interview with a male committee member).

### Superstition

Some women and men feared witchcraft particularly during the early months of pregnancy. A woman stated;

*During my first pregnancy, my mother- in- law instead that I should not disclose my pregnancy early even to the health care providers. She even informed my parents in the nearby village the risk I might face if people knew my status. There is nothing more they fear than prevalent witchcraft in this village (Interview* with a woman in Kilolo District).

The villagers still believe that care is only needed when women are pregnant until when their bellies become visibly big. As a result, emphasis is more on *how pregnant women should be careful about witches instead of attending ANC early or completing recommended visits.*

### Partner accompany policy

As part of prevention of mother-to-child transmission of HIV/AIDS, couples are required to attend the first ANC together in order to undergo HIV screening. In almost all FGDs women reported that if they attend ANC clinics for the first time without their partners, they are denied services. Female participants reported that this requirement increased the likelihood of many women to delay ANC attendance. This finding was also confirmed during stakeholders’ meetings, whereby almost all health care providers reported that if pregnant women went without partners, they were not attended to. Instead, they were instructed to request the company of their partners or otherwise present a letter from the village leaders in case their partners were not able to attend for some reasons. It was also found that some men tended to abandon their families especially when women become pregnant. This is affirmed in an interview with a health care provider:

*When many husbands in this area see that their wives are pregnant, they leave their home and seek casual labour elsewhere. Therefore, the wives have to keep waiting until their husbands come back because they cannot start the clinic alone. This was found to be a major challenge of the accompany policy in this area.* (Health care provider, Mufindi District).

Various factors were reported to discourage men from attending ANC with their partners (Table 1).

**Table 1:** Barriers to male participation in ANC

FGD with female participants	FGD with male participants
Men’s lack of trust. They sometimes believe that their partners have cheated	Multiple sexual relationships which make men doubt their involvement in pregnancy
Lack of knowledge of the importance of ANC	Lack of knowledge/understanding by men on matters pertaining to ANC
Men fear to attend ANC with their partners when they realize that the partners are pregnant within a short period after the last birth, and hence short birth spacing	Men fear to give company to their partners when they have failed to observe optimal birth spacing
Men fear HIV-screening, hence they depend on the results of their partners	Majority of men fear HIV-screening. They normally depend on the results of their partners
Customs and traditions among the Hehe and Bena still do not allow men to go with their partners for ANC	Traditions and customs among the Hehe and Bena still do not allow men to go with their partners for ANC

There was consensus among health care providers and some community members that denying services to women who attended ANC clinics without partners was important because it motivated men to attend ANC and screen for HIV. However, other participants in stakeholders’ meetings had the opinion that women were being punished for offences that were beyond their control.

### Rude language from health personnel

Rude language used by health care workers deter pregnant women to seek ANC on time. It was noted, for example, that nurses say that the constant complaints of pregnant women are nonsense as long as they receive their monthly salaries. Therefore, they should not expect them to behave the way women want them to. The views held by women resonated with that of men who stated that prevalence of harsh language affected not only their wives’ attendance, but also discouraged men to accompany their partners.

*Personally, I didn’t accompany my wife when she was pregnant recently. You know, at first we had a doctor who was very friendly; unfortunately, he was transferred to another facility. The new one, who is with us, discourages us from giving company to our wives. You feel so uneasy for disrespectful words when she attends to our wives* (FGD with men in Kilolo District)

Further investigation was done to explore the reasons for harsh language among health care providers. Heavy workload among them was one reason. Respondents also reported that health care providers staying in one station for a long time is a problem. Furthermore, failure of pregnant women to abide by given instructions incited health care providers to use impolite language.

### Shortage of health care providers

Acute shortage of health care providers in Kilolo and Mufindi districts affects effective provision of ANC. As a result, some pregnant women do not get ANC on time. Based on the 2014 staffing levels established by the Ministry of Health and Social Welfare, the minimum number of health care providers required is 15 for a dispensary and 39 for a health centre. There was consensus among participants of FGDs and interviews that health facilities were facing acute staff shortages (Table 2).

**Table 2:** Available and missing health care providers in the surveyed health facilities

Name of facility	Mazombe	Itungi	Ibumu	Nyololo	Igowole	Iramba	Kibengu	Kasanga	Ukumbi	Total
Proposed no of staff	15	15	15	15	15	15	15	39	15	**159**
Available Staff Percentage	5 33%)	5 (33%)	3 (20%)	2 (13.3%)	7 (46%)	6 (40%)	2 (13.3%)	21 (53%)	5 (33%)	**56 35%)**
Missing Staff	10 (67%)	10 (67%)	12 (80%)	13 (87%)	8 (54%)	9 (60%)	13 (87%)	18 (47%)	10 (67%)	**103 (65%)**

Source Filed Report April–June 2016

## Discussion

Socially constructed norms around gender continue to deter participation of men in pregnancy and childbirth. Men control decision-making at the household, which influences the timing of ANC attendance. Similarly, men control household economic resources which in turn influences women’s decision and ability to seek maternal and child health services. Men and the community still consider pregnancy as typically women’s domain, and they do not feel that they should be involved. These findings are consistent with previous studies [33–35]. Studies in Zimbabwe, Mozambique and Tanzania have discovered that women at an early stage of pregnancy delay ANC attendance in order to protect unborns against witchcraft and magical attacks from greedy neighbours [16, 26, 27].

There is a need for continued focus on education and sensitisation campaigns to the community on the importance of early ANC attendance. Educational interventions which target both men and women have been reported to increase health seeking behaviour among pregnant women and improve birth preparedness and complication readiness [36, 37]. However, despite their importance, most often, sensitisation campaigns are short-lived and thus they may not initiate enough change to enhance long-term behaviour change. It is important to note that these initiatives require massive mobilisation of human and financial resources [34]. In this contexts, there is need for health care workers and district health managers to empower community health workers to provide education and community sensitisation in their respective villages. In addition to home visits, education could be provided during various political and social gatherings in the community. In Tanzania, community health workers have been reported to play great roles in promoting male involvement in maternal and child health matters [38]. In addition, political, community and religious leaders should be sensitized and encouraged to provide health education to the community. Studies in other settings have revealed that the impact of traditional and religious leaders is significant when they are actively engaged in providing health education to the community [34, 35, 39].

In most health facilities it is mandatory for pregnant women to attend the first ANC along with their spouse. This is intended to ensure that couples are screened for HIV as part of the Prevention of Mother to Child Transmission (PMTCT) programme. The move is also meant to create an opportunity to provide education on danger signs during pregnancy, and on how to become birth-prepared and complication-ready [36]. Implementation of this policy, however, deters men from attending ANC mainly due to fear of HIV screening. As a result, women delayed ANC attendance and sometimes completely skipped ANC due to lack of partners’ support. This corroborates with other recent studies in Malawi, Rwanda and Tanzania [40–43]. The alternative solution of obtaining an approval letter from the village leaders was said to further delay ANC attendance and increased shame for women [42]. This may be especially true given the prevailing stigma associated with premarital pregnancies [42, 44]. Furthermore, men who go with pregnant women for ANC for the first time are not necessarily their authentic husbands or partners [42, 43]. While it is important for couples to attend ANC together, it is unfair to deny services women who attend alone. Instead of denying them services, health care workers should use friendly verbal and written invitations to encourage male men to accompany their partner as they attend ANC even after the first visit. Written and verbal invitations have been reported to increase participation of men in PMTCT in various settings [34, 45, 46]. Furthermore, in Malawi male champions and male gate keepers particularly village, community and religious leaders were identified and trained to deliver messages to other men in the community [35]. In our contexts, male champions could play a great role in educating men on family planning in order to address the problem of short birth spacing which is a factor to delay ANC.

### Strengths and limitations of the study

In our study data were collected from diversified respondents namely women, men and health care workers. This made it possible to triangulate the findings across different types of respondents. In addition, the initial findings were validated during stakeholders’ meetings. However, the study was conducted in only two rural districts and the findings may not adequately reflect experiences in other districts in Tanzania.

## Conclusion

In this study in the Southern Highlands of Tanzania traditional gender roles and cultural beliefs about pregnancy as well as health system factors continue to influence the timing of ANC attendance. Improving early ANC attendance, therefore, requires integrated interventions that address both community and health systems barriers. Health education on timing and importance of early antenatal care should be strengthened in the communities. Additionally, while spouse accompany policy is important for prevention of mother-to-child transmission, the implementation of the policy should not infringe women’s rights to access ANC, which may jeopardise maternal and neonatal outcome.

## Supplementary information

**Additional file 1.** Focus Group Guides.

**Additional file 2.** Interview Guide.

## Data Availability

The datasets generated and analysed during this study are not publicly available since participants did not give consent for public sharing of their information. However, summaries of the information are available from the corresponding author upon reasonable request. The interview and FGD guides for all study participants are also available upon request.

## Abbreviations

AIDS: Acquired Immune Deficiency Syndrome
ANC: Antenatal Care
FGD: Focus Group Discussion
HIV: Human Immunodeficiency Virus
IMCHA: Innovating for Maternal and Child Health in Africa
NGO: Non-Governmental Organization
PMTCT: Prevention of Mother to Child Transmission
WHO: World Health Organization
