# Supplementary material for: Why do pregnant women in Iringa region in Tanzania start antenatal care late? A qualitative analysis
Source: BMC Pregnancy Childbirth. 2020 Feb 24;20:126. doi: 10.1186/s12884-020-2823-4 (PMC7041254; doi:10.1186/s12884-020-2823-4)
Supplement: Supplementary file 1 — Additional file 1. Focus Group Guides. [file 12884_2020_2823_MOESM1_ESM.doc]

**Focus Group Discussion Guide for Men**

Good morning/afternoon, my name is ... …. and We are working for the **IMCHA** project. Currently we are conducting a survey on mother and child health in your community. The information will be useful for us, the government and other agencies in planning and delivery of health services. I want to start by thanking you for agreeing to participate. We are very interested to hear your valuable opinion on the health of mothers and children in this community.

I also wish to assure you that the information you give us is completely confidential, and we will not associate your name with anything you say. You may refuse to answer any question or withdraw from the study at anytime. However, we hope you can participate fully since your opinion and information are very important.

The group discussion will take approximately one hour and I wish to kindly ask all of you to actively participate in the debate.

Do you have any questions before we begin? Can I now open the discussion?

**Questions**

**A: Availability & Access to MNCH Services**

1. Which MNCH services are available to you? Are there any other services which you would like to receive?
2. Do you access/utilize/ these services?

**B: Distribution of Labour and Household Decision Making**

1. Who usually makes decisions about health care for women and children?
2. Who usually makes decisions about making major household purchases?
3. Who usually makes decisions about making purchases for daily needs?
4. Many factors prevent women from getting medical advice or treatment.  When women are sick and want to get medical advice or treatment, is each of the following a big problem or not?

- Getting permission to go
- The distance to a health facility
- Not wanting to go alone
- Availability of transport
- Cost of transport
- Road condition
- Availability of health providers
- Attitudes of the health providers
- Lack/poor facilities/equipment

**C: Decision on Place for Delivery**

1. The Ministry of Health encourages men to accompany their wives/partners for MCH services.

- What is your opinion about this?
- Is it common for men to accompany their wives/partners for MCH services?
- What are the factors which encourage/discourage men to accompany their wives/partners for MCH services?

1. Who takes the decision regarding the place of delivery? Do women have a say?
2. The Ministry of Health recommends that all women who are pregnant should start clinic not later than 12 weeks after getting pregnancy. What is your opinion about this? Why do some women start clinic late?

10. The Ministry of Health recommends that all women should give birth at health facilities. What is your opinion about this?

11. What are the driving factors influencing places of child birth in this community?

12. What is your opinion about the services provided by the TBAs?

13. Are there any strategies put in place to encourage facility deliveries?

**D: Cultural Practices/Belief Influencing MCH**

1. What are the cultural practices/beliefs which influence the health of mothers and children? Probe the following:

- Early booking (Kuanza Kliniki Mapema)
- Men to accompany their wives/partners to seek MCH services
- Management of cord (Kitovu cha mtoto)
- Early breast feeding
- Seeking MCH services outside the formal health system
- Deliver at home or at the TBAs.

1. What are your final thoughts and recommendations concerning MNCH?

**We have come to the end of the discussion. Thank you for participating actively**

**Focus Group Discussion Guide for Women**

Good morning/afternoon, my name is ... …. and We are working for the **IMCHA** project. Currently we are conducting a survey on mother and child health in your community. The information will be useful for us, the government and other agencies in planning and delivery of health services. I want to start by thanking you for agreeing to participate. We are very interested to hear your valuable opinion on the health of mothers and children in this community.

I also wish to assure you that the information you give us is completely confidential, and we will not associate your name with anything you say. You may refuse to answer any question or withdraw from the study at anytime. However, we hope you can participate fully since your opinion and information are very important.

The group discussion will take approximately one hour and I wish to kindly ask all of you to actively participate in the debate.

Do you have any questions before we begin? Can I now open the discussion?

**Questions**

**A: Availability & Access to MNCH Services**

1. Which MNCH services are available to you?
2. Do you access/utilize/ these services?
3. Do you need to travel to access these services? How far? How do you travel?
4. What are the treatment/advice hours for these services? Do you think the opening hour is adequate to meet my needs?
5. Do you receive adequate attention from the doctors and nurses?
6. How do you feel about time spent waiting to see a doctor?
7. How do you feel about time spent to discuss your health problems with the doctor?
8. Do the health providers treat you with respect and dignity?
9. Do the health providers treat you in privacy without being seen by others?
10. Do the health providers treat you in privacy without being heard by others?
11. How do you assess the cleanness of the health facility which you normally go for treatment?
12. How do you feel about the cost of the service/treatment received?
13. How do you feel about the overall quality of the service received at the health facility?

**B: Distribution of Labour and Household Decision Making**

1. Who usually makes decisions about health care for women and children?
2. Who usually makes decisions about making major household purchases?
3. Who usually makes decisions about making purchases for daily needs?
4. Many factors prevent women from getting medical advice or treatment for themselves.  When you are sick and want to get medical advice or treatment, is each of the following a big problem or not?

- Getting permission to go
- The distance to a health facility
- Not wanting to go alone
- Availability of transport
- Cost of transport
- Road condition
- Availability of health providers
- Kauli /Tabia za watoa hudumu za afya
- Lack/poor facilities/equipment

**C: Decision on Place for Delivery**

1. The Ministry of Health encourages men to accompany their wives/partners for MCH services.

- What is your opinion about this?
- Is it common for men to accompany their wives/partners for MCH services?
- What are the factors which encourage/discourage men to accompany their wives/partners for MCH services?

1. Who takes the decision regarding the place of delivery? Do women have a say?
2. The Ministry of Health recommends that all women who are pregnant should start clinic not later than 12 weeks after getting pregnancy. What is your opinion about this? Why do some women start clinic late?

21. The Ministry of Health recommends that all women should give birth at health facilities. What is your opinion about this?

22. What are the driving factors influencing places of child birth in this community?

23. What is your opinion about the services provided by the TBAs?

24. Are there any strategies put in place to encourage facility deliveries?

**D: Cultural Practices/Belief Influencing MCH**

1. What are the cultural practices/beliefs which influence the health of mothers and children? Probe the following:

- Early booking (Kuanza Kliniki Mapema)
- Men to accompany their wives/partners to seek MCH services
- Management of Kitovu cha mtoto
- Early breast feeding
- Seeking MCH services outside the formal health system
- Deliver at home or at the TBAs.

1. What are your final thoughts and recommendations concerning MNCH?

**We have come to the end of the discussion. Thank you for participating actively**
