# Supplementary material for: Why do pregnant women in Iringa region in Tanzania start antenatal care late? A qualitative analysis
Source: BMC Pregnancy Childbirth. 2020 Feb 24;20:126. doi: 10.1186/s12884-020-2823-4 (PMC7041254; doi:10.1186/s12884-020-2823-4)
Supplement: Supplementary file 2 — Additional file 2. Interview Guide. [file 12884_2020_2823_MOESM2_ESM.doc]

**Interview guide for Healthcare Workers**

**Questions**

**1. Profile of Health Service Provider**

Age (optional); Sex, Educational qualification; Professional training; Designation; Years of work experience

**2. Health sectors response to maternal and child health issues**

a. What are the MNCH services being provided in the health facilities in this district?

- Comment on the maternal mortality in this district? What factors explain this state?
- Comment on the child mortality in this district? What factors explain this state?

b. Are the MNCH services offered by the health facilities adequate? What are lacking?

c. What is the capacity of the health care system to provide MNCH services?

- Probe for Human resources; availability of resources/commodities/infrastructures

d. What are the challenges faced by the health facilities in dealing with MNCH?

e. How do the health facility address these challenges?

f. Are there established and functioning referral systems for MNCH services?

g. What are the existing monitoring and evaluation tools for MNCH services?

**3. Community related factors influencing provision and uptake of MNCH services**

1. Who accompanies women to health appointments? Is it common for husbands to accompany their wives? If not, why?
2. Who takes children to the health clinic when they are sick?
3. What cultural beliefs influence the MNCH?
4. What are the driving factors influencing places of child birth in this community?
5. What is the level of ANC uptake among women of reproductive age in this district?
6. In your opinion, which factors contribute to late initiation of ANC by pregnant women in this area?
7. What should be done to ensure pregnant women start ANC early?

**Thanks you for participating and sharing your views**
